# Association between exposure to radioactive iodine after the Chernobyl accident and thyroid volume in Belarus 10-15 years later

**DOI:** 10.1186/s12940-021-00820-0

**Published:** 2022-01-07

**Authors:** Ekaterina Chirikova, Robert J. McConnell, Patrick O’Kane, Vasilina Yauseyenka, Mark P. Little, Victor Minenko, Vladimir Drozdovitch, Ilya Veyalkin, Maureen Hatch, June M. Chan, Chiung-Yu Huang, Kiyohiko Mabuchi, Elizabeth K. Cahoon, Alexander Rozhko, Lydia B. Zablotska

**Affiliations:** 1 Department of Epidemiology and Biostatistics, School of Medicine, University of California, San Francisco, 550 16th Street, San Francisco, CA 94158 USA; 2 The New York Thyroid Center, Columbia University, New York, NY USA; 3 Department of Radiology, Thomas Jefferson University Hospital, Philadelphia, PA USA; 4 Republican Research Center for Radiation Medicine and Human Ecology, Gomel, Belarus; 5 Radiation Epidemiology Branch, Division of Cancer Epidemiology and Genetics, National Cancer Institute, Bethesda, MD USA; 6 Institute for Nuclear Problems, Belarusian State University, Minsk, Belarus

**Keywords:** Chernobyl nuclear accident, Radioactive iodine, Radiation, Thyroid gland, Thyroid volume, Thyroid pathology, Dose-response relationship, Environmental exposure

## Abstract

**Background:**

While there is a robust literature on environmental exposure to iodine-131 (^131^I) in childhood and adolescence and the risk of thyroid cancer and benign nodules, little is known about its effects on thyroid volume.

**Methods:**

To assess the effect of ^131^I dose to the thyroid on the volume of the thyroid gland, we examined the data from the baseline screening of the Belarusian-American Cohort Study of residents of Belarus who were exposed to the Chernobyl fallout at ages ≤18 years. Thyroid dose estimates were based on individual thyroid activity measurements made shortly after the accident and dosimetric data from questionnaires obtained 10-15 years later at baseline screening. During baseline screening, thyroid gland volume was assessed from thyroid ultrasound measurements. The association between radiation dose and thyroid volume was modeled using linear regression where radiation dose was expressed with power terms to address non-linearity. The model was adjusted for attained age, sex, and place of residence, and their modifying effects were examined.

**Results:**

The analysis was based on 10,703 subjects. We found a statistically significant positive association between radiation dose and thyroid volume (*P* < 0.001). Heterogeneity of association was observed by attained age (*P* < 0.001) with statistically significant association remaining only in the subgroup of ≥18 years at screening (*P* < 0.001). For this group, increase in dose from 0.0005 to 0.15 Gy was associated with a 1.27 ml (95% CI: 0.46, 2.07) increase in thyroid volume. The estimated effect did not change with increasing doses above 0.15 Gy.

**Conclusions:**

This is the first study to examine the association between ^131^I dose to the thyroid gland and thyroid volume in a population of individuals exposed during childhood and systematically screened 10-15 years later. It provides evidence for a moderate statistically significant increase in thyroid volume among those who were ≥ 18 years at screening. Given that this effect was observed at very low doses and was restricted to a narrow dose range, further studies are necessary to better understand the effect.

## Background

Risks associated with radioactive iodine are a public health concern both because of its widespread clinical application for treatment of thyroid conditions and the fact that it is preferentially absorbed and accumulated by the thyroid after nuclear facility accidents [1]. The accident at the Chernobyl nuclear power plant in Ukraine in 1986 resulted in the exposure of a large population to high doses of radionuclides, primarily ^131^I. It raised concerns about the increased risk of thyroid pathologies in children whose growing thyroid is particularly sensitive to this kind of exposure [2, 3]. Findings from two cohorts of children residing in the contaminated territories of Belarus and Ukraine at the time of the accident and screened for thyroid pathologies at least every 2 years demonstrated a dose-response association between ^131^I thyroid dose and thyroid cancer and follicular adenoma, and also presented evidence of increased risk of hypothyroidism [4–10]. However, little is known about the effects of ^131^I thyroid dose on thyroid volume enlargement which could reflect a transitional state that may progress to overt forms of thyroid diseases (e.g., diffuse or nodular goiter).

To our knowledge, no studies have examined the dose-response association between ^131^I thyroid dose and thyroid volume. Increased thyroid volume is an essential part of simple nontoxic goiter or diffuse goiter diagnoses, and several studies examined the risk of these conditions after exposure to radioiodine. However, the majority of these studies lacked individual measurements of dose to thyroid [11–16], and the few studies that had thyroid dose measurements had contradictory findings [17, 18]. Understanding the dose-response relationship between ^131^I exposure and thyroid volume will inform clinical decisions and public health interventions targeted at individuals exposed to radioactive iodine as a result of occupational exposure, medical treatment, or nuclear fallout.

Two parallel screening cohort studies of children exposed to the fallout from Chernobyl were established in Belarus and Ukraine to examine associated health risks. Standardized approach was used in screening of these two cohorts that included ultrasound measurements for all subjects. In this study we assessed the long-term effect of exposure to ^131^I after the Chernobyl accident on the volume of the thyroid gland 10-15 years later by examining baseline data from the cohort in Belarus.

## Methods

### Study subjects

We used baseline data from the Belarusian-American Cohort Study (referred to as the “BelAm” further in the text) conducted by the Chornobyl Thyroid Diseases Study Group of Belarus, Ukraine, and the USA and funded by the U.S. National Cancer Institute. A detailed description of the BelAm study population and methods is provided elsewhere [4, 19]. Briefly, the source population of the BelAm cohort included individuals who were born between April 26, 1968 and April 25, 1986, resided in Belarus at the time of the accident, and had their ^131^I thyroid activity measured in 1986 within 2 months after the accident. Out of 38,543 records in the dose file for the source population, 16,213 individuals, who were eligible for the study and who could be traced, were contacted. Among these eligible individuals, 11,970 were medically examined at the baseline screening in 1996-2004 (11,903 in 1996-2001, 67 in 2002-2004). Baseline screening included collection of blood and urine samples, thyroid palpation, ultrasound examination, collection of medical history, dosimetry interview, and fine-needle aspiration upon reference [19].

Later, 238 records were excluded due to incorrect identification (*n* = 9), out of range age (*n* = 114), and inadequate thyroid dose estimate (*n* = 115), thus leaving 11,732 records available for analysis. For the purposes of this study, we excluded a few additional categories of individuals: (1) those whose thyroid volume was not measured at the baseline screening (*n* = 163); (2) those who had a confirmed thyroid cancer, nodular goiter, thyroid surgery, or aplasia (*n* = 703); (3) those who had doses over 5 Gy (*n* = 161); (4) and those with extreme values of thyroid volume (over 80 ml, *n* = 2). Diagnoses of thyroid cancer and nodular goiter were confirmed via fine-needle aspiration biopsy. Individuals with thyroid cancer, nodular goiter, thyroid surgery, or aplasia were excluded because thyroid volume could be increased from these conditions. Individuals with doses over 5 Gy were excluded as outliers. As a result, the analytical sample comprised 10,703 individuals.

This study was exempted from the institutional review board of the University of California, San Francisco since it only used existing de-identified data. The original BelAm cohort study was approved by institutional review boards in Belarus and the United States. Informed consent was provided by the study participants or by accompanying guardians for minors.

### Measures

Thyroid volume was assessed for all participants at baseline screening via ultrasound examination. It was carried out by physicians with special training and expertise in thyroid ultrasonography. Thyroid gland volume was automatically calculated on the basis of its three dimensions (length, width, depth) and measured in ml based on the volume of an ellipsoid (length x width x depth × 0.479) [20]. All participating personnel were blinded to the subjects’ individual radiation dose.

Individual dose of ^131^I to the thyroid gland, which represented about 92% of the thyroid dose [21] and came mainly from the consumption of contaminated milk, was measured in mGy and reconstructed from three sources: (1) estimated ^131^I activity in the thyroid derived from direct thyroid measurements of each cohort member that were obtained in April-June 1986, (2) application of the radioecological model used to account for temporal variation of ^131^I intake, (3) interview data on dietary and lifestyle habits (e.g. contaminated milk consumption) obtained during screening. Parameters of the dosimetry model were updated using additional sources, such as thyroid volume measurements done by the Sasakawa Memorial Health Foundation [22] used to derive age-specific thyroid masses typical for the Belarusian population; measurements of ^131^I in soil, grass, and cow’s milk [23] used to verify the validity of the calculated ^131^I deposition density in the settlements and to derive the parameters of the dose reconstruction model. The arithmetic mean of 1000 individual stochastic doses due to ^131^I intake, calculated for each cohort member, was used in this study as the thyroid dose from ^131^I [24].

Factors potentially associated with thyroid dose and thyroid volume, such as sex, age and oblast of residence at baseline screening (oblast is an administrative subdivision similar to a state or province), urban/rural status of residence, spot urinary iodine concentration, dietary intake of iodine, smoking habit, thyroid hormone levels, and family history of thyroid pathologies, were obtained for all participants via personal interviews and lab tests. Age and oblast of residence were also recorded at the time of the accident when ^131^I activity in thyroid was measured.

### Statistical analysis

The relationship between ^131^I dose to thyroid and thyroid volume was analyzed using multivariable linear regression models. To reflect the non-linear effect of ^131^I dose on thyroid volume, we considered multiple power terms in a form: *dose*^1/*k*^, where k takes the values 1, 2, 4, 8, 16, 32, 64. We used step Akaike Information Criterion [25] to determine the number of power terms for dose to be included in the regression model. Risk factors such as sex, age and oblast of residence at baseline screening were a priori included in the model as covariates since their role in the relationship between thyroid dose and thyroid pathologies was demonstrated in previous studies of this cohort and is justified by biological plausibility [4, 8]. Age at screening was included in the model in a form of linear and quadratic terms to address non-linearity and centered around the median. Oblast of residence was used as the primary proxy indicator of iodine deficiency, which can increase the absorbed ^131^I dose, modify the dose-response, and is a strong predictor of thyroid enlargement [26–29]. Iodine intake at the time of the accident and changes in iodine nutrition after the accident varied by oblast [30], making it a useful indicator of iodine deficiency both at the time of the accident and at screening.

Additionally, we examined other potential confounding variables by sequentially adding them to the model and evaluating change in the overall effect of dose and model fit. Potential confounders include urban/rural status of residence, smoking, usage of multivitamins, iodine supplements or iodized salt, level of urinary iodine via urinary iodine concentration, thyroid-stimulating hormone (TSH) level, and family history of thyroid cancer and other thyroid pathologies. These covariates were retained in the model if they satisfied two criteria, including improved model fit based on the lower value of the Akaike Information Criterion and change in the overall effect of dose by more than 10%.

Effect modification by age, sex, and oblast of residence at the time of the accident and at baseline screening was also examined. Given the association of age with the volume of thyroid gland, the analysis was also conducted separately by age groups. The cut point at 18 years was chosen because thyroid gland is generally considered to stop growing after that age and widely used adult thyroid volume norms were estimated for those 18 and older [31]. To confirm this theoretical assumption, we conducted a sensitivity analysis varying the cut point to be at age 16, 18, 20, 22, or 24, and evaluating heterogeneity in the effect of dose in the two age subgroups. The first cutpoint to show statistically significant heterogeneity was age 18 years old at screening. Consequently, the volume of the thyroid gland overall and in the two subgroups was given by the following:

$$Thyroid\ Volume={\beta}_0+{\beta}_1D+{\beta}_2{D}^{1/2}+{\beta}_3{D}^{1/4}+{\beta}_4M+{\beta}_5O1+{\beta}_6O2+{\beta}_7A+{\beta}_8{A}^2+\varepsilon,$$

where D denotes ^131^I dose to thyroid gland, M denotes gender male (female is a reference), O1 and O2 denote oblasts of residence at screening (three categories, Minsk is a reference, Gomel, and Other are O1 and O2 correspondingly), A1 denotes age at screening centered around the median, and *ε* denotes random error.

When reporting thyroid volume distribution among study participants, common cut-offs for thyroid enlargement in iodine-sufficient regions were applied, such that thyroid glands > 18 ml in females and > 25 ml in males were considered enlarged [31].

STATA (version 16.1) was used for all data analyses [32]. All statistical tests were two-sided with a pre-specified type I error rate of 0.05.

## Results

The study sample consisted of 10,703 individuals, 51% of whom were female (Table 1) and the average age at the time of the accident was 8 years (standard deviation (SD) = 5 years, minimum 0, maximum 18 years, data not shown). More than half of the study participants resided in the closest proximity to Chernobyl, in Gomel oblast, both at the time of the accident (73%, data not shown) and at the time of screening (61%, Table 1). At the time of screening, 65% of study participants (Table 1) had some degree of iodine deficiency (defined as urinary iodine levels < 100 mcg/L, following the WHO classification of iodine nutrition status [33]). Thyroid dose among participants ranged from 0.0005 to 5 Gy with a mean of 0.54 Gy (SD = 0.74 Gy) and median of 0.26 Gy (data not shown).

**Table 1:** Characteristics of thyroid volume overall and by subgroups, Belarus, 1996-2004, total *N* = 10,703

	Overall, n (%)	Thyroid volume (ml)					*P*-value^a^
		Mean	Median	95% CI	IQR		
**Overall**	10,703 (100)	12.9	12.2	12.9, 13.0	5.5		
**Sex**							< 0.001
Female	5472 (51)	12.0	11.4	11.9, 12.1	4.5		
Male	5231 (49)	13.9	13.3	13.8, 14.1	6.2		
**Age at screening (years)**							< 0.001
10-17	3312 (31)	10.5	10.0	10.4, 10.6	4.4		
18-33	7391 (69)	14.0	13.2	13.9, 14.1	5.5		
**Oblast of residence at screening**							< 0.001
Minsk	2973 (28)	13.1	12.3	12.9, 13.3	5.5		
Gomel	6522 (61)	12.6	11.9	12.5, 12.7	5.3		
Other^b^	1208 (11)	14.6	13.7	14.2, 14.9	6.5		
**Urban/rural status**							0.096
Urban	6537 (61)	12.8	12.1	12.7, 13.0	5.8		
Rural	4166 (39)	13.1	12.4	13.0, 13.3	5.4		
**Urinary iodine concentration**^**c**^ **(mcg/L)**							0.002
< 20	1156 (11)	13.5	12.7	13.2, 13.8	5.9		
20-49	2781 (26)	13.0	12.2	12.8, 13.2	5.6		
50-99	2993 (28)	12.9	12.2	12.7, 13.0	5.6		
≥ 100	3773 (35)	12.8	12.0	12.6, 12.9	5.4		

*Abbreviation*: *CI* confidence interval, *IQR* interquartile range

^a^*P*-values from the Kruskal-Wallis test

^b^Includes Brest, Grodno, Mogilev, and Vitebsk oblasts

^c^WHO criteria for iodine deficiency: < 20 severe, 20-49 moderate, 50-99 mild, ≥100 not deficient [33]

**Fig. 1:**
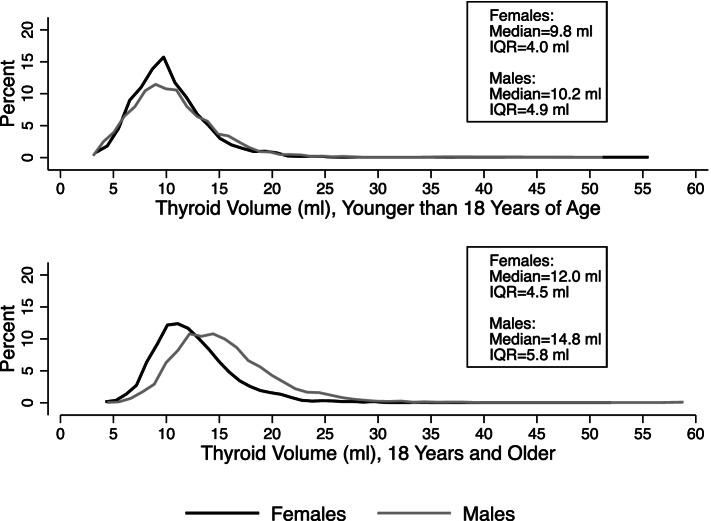
Distribution of thyroid volume by age and sex subgroups. IQR – interquartile range

Thyroid volume in the study ranged from 2.8 to 56.1 ml with a mean of 12.0 ml (SD = 4.1, 95% CI: 11.9, 12.1) among females and from 2.6 to 59.4 ml with a mean of 13.9 ml (SD = 5.3 ml, 95% CI: 13.8, 14.1) among males. Mean thyroid volume was significantly higher for males vs. females, older vs. younger individuals, those residing in oblasts other than Minsk or Gomel, and having lower urinary iodine concentration. (Table 1). There were 395 females (7%) and 163 males (3%) in the study whose thyroid volume would be considered enlarged (over 18 ml for females and over 25 ml for males [31], data not shown). Although the distribution of thyroid volume was very similar in males and females who were younger than 18 years at screening, it differed by sex in older individuals with a fully-grown thyroid (18 years and older at screening) (Fig. 1).

In the total sample the association between thyroid dose and thyroid volume was statistically significant (*P* < 0.001, Table 2). The association was not linear. Using a power model and after adjusting for age, sex, and oblast at screening, we observed an increase in thyroid volume in the low dose range and flattening of the effect for doses above 0.15 Gy. Predicted thyroid volume for dose 0.15 Gy was 1.30 ml (95% CI: 0.64, 1.96) larger than thyroid volume for dose 0.0005 Gy (lowest dose in the cohort). This effect did not change for larger doses, so that the predicted thyroid volume for dose 1 Gy was 1.33 ml (95% CI: 0.73, 1.93) larger than the thyroid volume for dose 0.0005 (Table 2). When we included observations with doses 5-39 Gy in the analysis, we observed an attenuation of effect (data not shown). Although our recent analysis of records for patients with high dose estimates confirmed, with a high degree of confidence, the reliability of thyroid doses exceeding 5 Gy [34], all further analyses excluded these observations due to their small number (*n* = 161) and the possibility that these may be determined by completely different tissue reaction mechanisms [35].

**Table 2:** Thyroid volume (ml) estimates with 95% confidence intervals from the linear regression models, Belarus, 1996-2001

Explanatory parameter	Thyroid volume estimates		
	Overall (*N* = 10,703)	Younger than 18 years at screening (*n* = 3312)	18 years or older at screening (*n* = 7391)
**Radiation**^**a**^			
0.0005 Gy^b^	Reference	Reference	Reference
0.001 Gy	0.129 (0.050, 0.208)	0.120 (−0.016, 0.256)	0.123 (0.024, 0.222)
0.05 Gy	1.073 (0.488, 1.658)	0.956 (−0.080, 1.992)	1.035 (0.316, 1.754)
0.1 Gy	1.225 (0.584, 1.866)	1.075 (−0.073, 2.222)	1.190 (0.407, 1.972)
0.15 Gy	1.297 (0.637, 1.957)	1.125 (−0.065, 2.314)	1.266 (0.464, 2.067)
0.5 Gy	1.385 (0.742, 2.027)	1.144 (−0.041, 2.330)	1.390 (0.620, 2.159)
1 Gy	1.326 (0.728, 1.925)	1.057 (−0.053, 2.166)	1.375 (0.660, 2.089)
2 Gy	1.206 (0.614, 1.798)	0.938 (−0.126, 2.003)	1.323 (0.589, 2.056)
4 Gy	1.135 (0.234, 2.035)	0.950 (−0.435, 2.335)	1.352 (0.091, 2.612)
4.5 Gy	1.146 (0.114, 2.178)	0.990 (−0.550, 2.530)	1.383 (−0.084, 2.851)
	*P < 0.001*^*c*^	*P = 0.247*^*c*^	*P < 0.001*^*c*^
**Sex**			
Female	Reference	Reference	Reference
Male	2.052 (1.892, 2.213)	0.369 (0.120, 0.617)	2.779 (2.578, 2.980)
	*P < 0.001*	*P = 0.004*	*P < 0.001*
**Oblast at screening**			
Minsk	Reference	Reference	Reference
Gomel	−1.230 (−1.444, −1.016)	− 1.094 (− 1.404, −0.784)	−1.223 (− 1.502, −0.945)
	*P < 0.001*	*P < 0.001*	*P < 0.001*
Other^d^	0.680 (0.385, 0.976)	0.689 (0.216, 1.162)	0.733 (0.365, 1.102)
	*P < 0.001*	*P = 0.004*	*P < 0.001*
**Age at screening (years)**			
Group median age^e^	Reference	Reference	Reference
1 year increase	0.380 (0.365, 0.396)	0.845 (0.758, 0.931)	0.235 (0.208, 0.261)
2 years increase	0.714 (0.683, 0.746)	1.495 (1.269, 1.721)	0.455 (0.401, 0.509)
5 years increase	1.437 (1.346, 1.529)	–	1.032 (0.847, 1.217)
7 years increase	1.687 (1.533, 1.840)	–	1.346 (1.012, 1.681)
	*P < 0.001*	*P < 0.001*	*P < 0.001*

^a^Holding all covariates at their reference values, the mean estimated thyroid volume at reference dose 0.0005 Gy (the lowest dose in the study) was 11.0 ml overall, 9.0 ml in the subgroup < 18 years, and 11.5 ml in the subgroup ≥18 years. Thyroid volume estimates at specific doses are an increase above the reference thyroid volumes

^b^Lowest dose in the study is 0.0005 Gy

^c^*P*-value for the association between thyroid dose and thyroid volume using linear regression model with three power terms for dose (*dose*^*1/1*^ *+ dose*^*1/2*^ *+ dose*^*1/4*^)

^d^Includes Brest, Grodno, Mogilev, and Vitebsk oblasts

^e^Median age (reference): 21 years overall, 15 years in the subgroup < 18 years, and 24 years in the subgroup ≥18 years

**Fig. 2:**
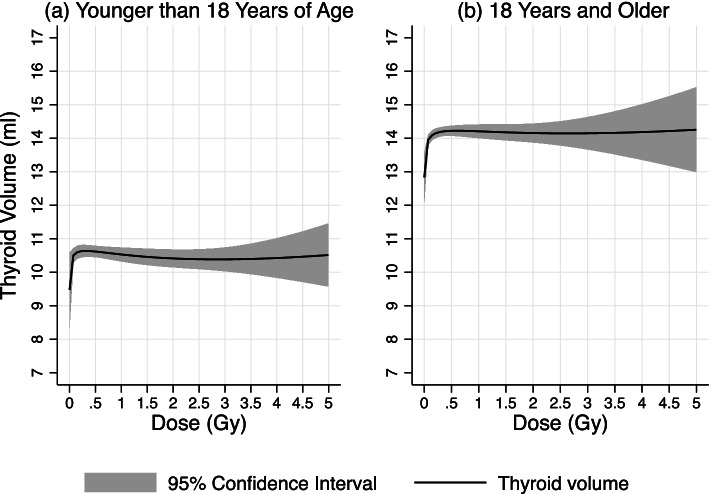
Association between thyroid dose due to ^131^I and thyroid volume at screening. Average effect of thyroid dose due to ^131^I on thyroid volume at screening from a linear regression model adjusted for sex, oblast, and age at screening in the subgroup of **a** younger than 18 years of age at screening; **b** 18 years and older at screening

Age at screening < 18 or ≥ 18 years was a significant modifier of the dose-response (*P* < 0.001, data not shown), so further results are presented within the two age subgroups. For those younger than 18 years at screening, the association between thyroid dose and thyroid volume was not statistically significant (*P* = 0.25, Table 2) after adjusting for age, sex, and oblast at screening. The fitted model explained 18% of variability in the data (R^2^ = 0.18). The observed effect of dose was smaller than in the total sample and not statistically significant. But the shape of the association between dose and thyroid volume had the same curvature as in the total sample, such that an increase at low doses was followed by flattening of the curve at higher doses (Fig. 2). Predicted thyroid volume for dose 0.15 Gy was 1.13 ml (95% CI: − 0.07, 2.31) larger than thyroid volume for dose 0.0005 Gy (lowest dose in the cohort). Predicted thyroid volume for larger doses was of the same order of magnitude (Table 2). In this age subgroup, in the model adjusted for thyroid dose, thyroid volume was significantly larger in males in comparison to females, in Brest, Grodno, Mogilev, and Vitebsk oblasts in comparison to Minsk or Gomel oblasts, and it increased with age (Table 2). No significant effect modification by sex (*P* = 0.22, data not shown), or oblast at the time of the accident (*P* = 0.75, data not shown) or at screening (*P* = 0.48, data not shown) was observed within a stratum of younger than 18 years of age at screening.

For those 18 years or older at the time of screening, we observed a statistically significant association between dose and thyroid volume (*P* < 0.001) after adjusting for age, sex, and oblast at screening. The fitted model explained only 14% of variability in the data (*R*^*2*^ = 0.14). The shape of the relationship followed the same curve as in the total sample and subgroup of < 18 years old at screening (Fig. 2). Predicted thyroid volume for dose 0.15 Gy was 1.27 ml (95% CI: 0.46, 2.07) larger than thyroid volume for dose 0.0005 Gy (lowest dose in the cohort). The effect was very similar for larger doses, so that predicted thyroid volume for dose 1 Gy was 1.38 ml (95% CI: 0.66, 2.09) larger than the thyroid volume for dose 0.0005 (Table 2). Similar to those < 18 years old, in the model adjusted for thyroid dose, thyroid volume was significantly larger in males in comparison to females and in Brest, Grodno, Mogilev, and Vitebsk oblasts in comparison to Minsk and Gomel oblasts (Table 2). No significant effect modification by sex (*P* = 0.33, data not shown), or oblast at the time of the accident (*P* = 0.23, data not shown) or at screening (*P* = 0.70, data not shown) was observed within a stratum of ≥18 years of age at screening.

## Discussion

This is the first analysis of baseline data from a cohort study in Belarus to examine the association between the dose to the thyroid gland and thyroid volume. We found that in children exposed to the Chernobyl fallout, 10-15 years later there was a statistically significant association between thyroid dose and increased thyroid volume, but only among those older than 18 years of age during screening. We observed a dose response that increased from 0.0005 to 0.15 Gy and then flattened with little further effect on thyroid volume at larger doses up to 5 Gy.

The reason for the observed effect is unclear. It is possible that this association is mediated by changes in TSH [9, 36]. According to this hypothesized biological mechanism, radiation dose to the thyroid gland could cause cell killing and decrease in thyroid hormones that leads to stimulation of the pituitary gland [37], and consequent increase in thyroid gland volume [38–40]. While associations between TSH level and thyroid volume [38–40] and between thyroid radiation dose and TSH [9, 36] are well described, the association between thyroid radiation dose and thyroid volume has not been previously examined. Our study adds to the existing literature by suggesting a highly non-linear effect of ^131^I dose on increased thyroid gland volume. We observed similar non-linear dose-response when we analyzed the association between ^131^I thyroid dose and prevalent hypothyroidism (serum TSH > 4 mIU/L) during baseline screening in this cohort [9]. At doses < 5 Gy, there was a similar sharp increase in odds ratio for the dose category 0.1-0.25 Gy, with no further changes with increasing doses. A different pattern was observed in a similar cohort in Ukraine, where a sharp increase in odds ratio for the dose category 0.1-0.25 Gy was followed by a linear dose-response for doses < 5 Gy [10]. Analysis of TSH on a continuous scale was also performed in Belarusian cohort but not in Ukrainian, and in contrast to analysis of TSH on a dichotomous scale (i.e. hypothyroidism) it demonstrated a significant positive dose-response with ^131^I for the whole range of doses up to 5 Gy [9]. It is somewhat unlikely that increase in thyroid volume at only the lowest doses observed in our study reflects an actual physiological effect. Several analyses of thyroid outcomes in this cohort observed flattening of the dose-response at doses > 5 Gy [4, 8, 41]. Similar effects were observed in studies of atomic bomb survivors and patients treated with radiotherapy, suggesting cell killing happening at very high doses [1, 42, 43]. However, this effect could be very different from the tissue expansion effect we examined in our study.

Our findings also shed light on the differing results from previous studies on the effect of ^131^I exposure on benign thyroid conditions, such as enlarged thyroid or diffuse goiter. Given that the effect we observed was highly non-linear and seen only at the lowest doses, it could easily be overlooked when specific thresholds for thyroid volume are applied to make the diagnosis of enlarged thyroid or diffuse goiter. The fact that some studies showed no effect [12, 17], while others found a positive association between ^131^I exposure and diffuse goiter [13, 18] is therefore unsurprising, and may also reflect low statistical power in some of these studies. Because our study focuses solely on thyroid volume rather than on a diagnosis of thyroid enlargement or diffuse goiter, we were able to detect even weak signals in the data that might otherwise be obscured.

One of the main advantages of our study is the availability of individual ^131^I doses to the thyroid for each participant in the cohort, which have been improved over time to account for possible uncertainties [21, 24]. This feature, along with a wide range of doses and the large and diverse cohort exposed to the Chernobyl fallout, contribute to the statistical power of this study. Another advantage is that thyroid volumes were measured for all study participants regardless of clinical presentation and according to a standardized protocol.

Some possible limitations of this study should be noted. One of them is that screening, including thyroid volume measurement, was conducted at least 10 years after the exposure. Missing data on dietary iodine during this period could lead to residual confounding from using proxy indicators of iodine nutrition status. As shown in previous studies, iodine deficiency could play an important role in the relationship between ^131^I dose to thyroid and pathologies closely related to thyroid volume, such as diffuse goiter [11, 26, 27, 30]. A negative association between urinary iodine excretion levels and prevalence of goiter was observed in several studies [11, 30]. There is also literature suggesting an increase in the absorbed ^131^I thyroid dose in iodine-deficient individuals [26–28]. Thus, having a proper measurement of iodine nutrition status on an individual level is important for estimating the direct effect of ^131^I dose. Since urinary iodine concentration at one time point is not an adequate measure of individual iodine status [44], we used oblast of residence as the primary proxy indicator of iodine nutrition status. Epidemiological studies and surveys of stable iodine in soil in settlement at the time of the accident demonstrate that iodine nutrition varied by oblast, thus making it a useful indicator of iodine deficiency [29, 30]. Although iodine deficiency is thought to increase ^131^I uptake by the thyroid gland [26], in our study we did not observe effect modification of dose by either oblast at the time of the accident, or at screening.

The fact that approximately 30% of the study population were under the age of 18 years at the time of screening and likely did not have a fully grown thyroid limits our ability to make inferences about this age subgroup. This issue is partially addressed by presenting stratum-specific effect estimates. Analyses using the data from the next screening cycles of the cohort should be able to address this limitation.

Since only 31% (11,918 of 38,543) of the source population participated in the BelAm study because of problems with tracing (*n* = 20,526), eligibility (*n* = 1804), or non-response (*n* = 4295) there is a potential for selection bias. There appeared to be a greater proportion of individuals from more severely contaminated areas and younger children (0-9 years old) in the cohort in comparison to the source population of individuals who were selected for tracing and recruitment to the study [19]. However, it is unlikely that individuals were selected differentially based on both dose and thyroid volume. Also, the study provides data on a large range of ^131^I thyroid doses (0.0005-5 Gy) and thyroid volumes (2.6-59.4 ml).

Given the unexpected non-linearity in radiation effect on the thyroid volume observed at the lowest doses for those ≥18 years old at screening, potential measurement errors should be considered. However, ultrasound measurements of thyroid volume were done by physicians with special training in ultrasound sonography who were blinded to the subjects’ individual radiation dose. The current study uses individual doses that are based on means of 1000 Monte Carlo simulations which take into account a number of uncertainties from the dose model. Therefore, potential systematic measurement errors, if any, were minimized.

## Conclusions

In summary, this is the first study to examine association between ^131^I dose to the thyroid gland and thyroid volume in a population of individuals exposed during childhood and systematically screened 10-15 years later. Previous studies had much more limited information on thyroid volume [18] or dose [11–15]. Our study adds to the current literature by providing evidence for a moderate statistically significant increase in thyroid volume among those who were 18 years or older 10-15 years after the accident. This increase in thyroid volume occurs only at very low ^131^I doses (0.0005-0.15 Gy), and volume does not show any further change with increasing radiation dose up to 5 Gy. Although it could be hypothesized that this effect is mediated by TSH levels, the established knowledge on the cell killing that occurs at much higher ^131^I doses prevents us from stating a clear biologic mechanism of the observed effect. Replication of this analysis using the data from the parallel study in Ukraine would help to better understand these findings. Extended follow-up is necessary to determine whether thyroid gland growth continues beyond the first 10-15 years after exposure. Since only part of this cohort was 18 years or older at the time of baseline screening, analysis of data from future screening cycles is needed to shed more light on whether there are any radiation effects on thyroid volume among those exposed at young ages.

## Data Availability

The datasets used and analyzed during the current study are not publicly available due to existing agreements between collaborators. Interested investigators should submit a request for data with a detailed analytical plan to the corresponding author and it will be reviewed by all collaborators.

## Abbreviations

^131^I: Iodine-131
BelAm: Belarusian-American Cohort Study
SD: Standard deviation
CI: Confidence interval
IQR: Interquartile range
